# Update on the Accuracy of Conventional and Digital Full-Arch Impressions of Partially Edentulous and Fully Dentate Jaws in Young and Elderly Subjects: A Clinical Trial

**DOI:** 10.3390/jcm11133723

**Published:** 2022-06-28

**Authors:** Maximiliane Amelie Schlenz, Julian Maximilian Stillersfeld, Bernd Wöstmann, Alexander Schmidt

**Affiliations:** Department of Prosthodontics, Dental Clinic, Justus Liebig University, Schlangenzahl 14, 35392 Giessen, Germany; julian.m.stillersfeld@dentist.med.uni-giessen.de (J.M.S.); bernd.woestmann@dentist.med.uni-giessen.de (B.W.); alexander.schmidt@dentist.med.uni-giessen.de (A.S.)

**Keywords:** clinical study, intraoral scanners, digital dentistry, impression techniques, full-arch impression, elderly population, dimensional measurement accuracy

## Abstract

To update the available literature on the accuracy of conventional and digital full-arch impressions using the latest hardware and software, participants of different age groups and dental status were investigated. An established reference aid-based method was applied to analyze five intraoral scanners (IOS) CS 3800 (CS), iTero Element 5D (IT), Medit i700 (ME), Primescan (PS), and Trios 4 (TR), and one conventional polyether impression (CVI). Forty-five participants were classified into three groups: Age 27.3 ± 2.7 years fully dentate, 60.6 ± 8.1 years fully dentate, and 65.7 ± 6.2 years partially edentulous. The IOS datasets were investigated using three-dimensional software (GOM Inspect), and plaster casts of CVI were analyzed using a co-ordinate measurement machine. The deviations of the reference aid to impressions were determined. No significant differences in age between the three groups were observed by the IOS in terms of trueness (*p* < 0.05). These findings were confirmed for precision, except for TR. In contrast to CS (mean ± standard deviation 98.9 ± 62.1 µm) and IT (89.0 ± 91.0 µm), TR (58.3 ± 66.8 µm), ME (57.9 ± 66.7 µm), and PS (55.5 ± 48.7 µm) did not show significant differences than those of CVI (34.8 ± 29.6 µm) in overall view. Within the study, the latest IOSs still showed limitations in the accuracy of full-arch impressions. However, they seemed to be unaffected by age and fully dentate or partially edentulous dentitions with small gaps.

## 1. Introduction

To date, a physical or virtual model of the intraoral situation is required for any indirect restoration or dental appliances [1]. Therefore, several conventional and digital techniques are currently available for full-arch impressions [2]. However, for impression-taking in the aged population, data are scarce [3]. In contrast to young, fully dentate patients, who mostly require impressions for orthodontic appliances or night guards, tooth loss and prosthodontic restorations are expected with increasing patient age. Furthermore, the demographic change leads to an elderly population with patients presenting a high number of natural teeth due to preventive dental hygiene concepts [4,5]. Thus, dentists are facing an aging population with increasing fixed-dental restorations (FDP). This topic needs to be addressed urgently.

Even though the general requirements for accuracy according ISO 5725-1 (mean values describing trueness, standard deviation (SD) describing precision) [6] are precise representations of the intraoral situation and an exact transfer to the extraoral model in this context, aged dentitions often exhibit attachment loss of the soft tissue with gingival recession and extensive interdental areas in contrast to young, natural dentate jaws and therefore present the practitioner with increased challenges [7,8]. Apart from the physiological aging of dentitions, the high prevalence of periodontitis, up to 42% in patients aged 40–60 years and up to 68% in patients aged >65 years, is also a contributing factor [9,10]. The implications of severe periodontitis are tooth loss, pathologic tooth migration with malocclusion, and flaring or elongation of teeth with bite deepening [11,12,13]. In summary, several undercuts complicate accurate impression taking.

A previous clinical study revealed that digital impressions with intraoral scanners (IOS) are superior to conventional polyvinyl siloxane impressions concerning the ability to display interdental areas in periodontally compromised dentitions in the aged population [3]. This can be explained by tearing and distortion of the conventional impression material during the removal of the impression because the elastomeric material flows into the undercuts and sets. However, the accuracies of both digital and conventional impressions were not investigated. For the entire impression of areas with undercuts, the scanning tip of the IOS cannot be positioned parallel to the tooth surface, resulting in angulations of up to 45°. Whether this angulation may cause inaccuracy in intraoral scan datasets needs to be discussed. A laboratory study by Desoutter et al. [14] has described higher noise for the IOS datasets captured with angulated surfaces of 30° and 45° than that with plane surfaces without angulation. To the best of the authors’ knowledge, no study has investigated the influence of aged dentition accompanied by further challenges on the accuracy of full-arch impressions.

Although clinical studies described superior accuracy for IOSs of short-span FDP within one quadrant compared to conventional impressions (CVIs), the latter still revealed the highest accuracy for long-span distances in the full-arch [15,16]. This is because the main problem with the IOS is that all scanning systems available in the market today do not allow an entire jaw or even just one-half of the jaw to be captured at once. All systems provide only sectional images covering a small area and must be merged by the scanner’s software in a matching/stitching process to create an overall model of the complete jaw. Although the original accuracy of the scanners is very high in the systems currently available in the market, these matching algorithms determine how accurately the overall system of hardware and software can map the geometry of the jaw. Matching errors lead to a steady increase without a compensable total error as the reconstruction of the jaw progresses along the scan path [17]. This is a fundamental disadvantage of the digital impression technique compared to the conventional methods because the latter captures the jaw all at once. Further development of hardware and software in recent years has shown a constant improvement in the IOS; hence, the latest IOS generations might overcome this limitation [16,18]. However, for new IOSs, such as CS 3800 (Carestream Dental, Atlanta, GA, USA), iTero Element 5D (Align Technology, San José, CA, USA), and Medit i700 (Medit, Seoul, South Korea), no clinical data for the accuracy of full-arch impression have been published yet.

To assess the accuracy of different impression techniques, a reference aid that displays the actual patient’s situation is indispensable [15]. Otherwise, only the respective deviations of different impression techniques can be examined. Only two reference aid-based methods have been described in the literature [19,20]. However, clinical data are only available for fully dentate jaws. Recently, Kontis et al. [21] published the first data on partially edentulous models based on a laboratory study with a reference aid, revealing a reduced accuracy compared to that of fully dentate models. In particular, edentulous areas in the mandible with mucosal mobility and saliva may be challenging in clinical impression taking.

Therefore, this clinical study aimed to update the available literature on the accuracy (trueness and precision according to ISO 5725 [6]) of conventional and digital full-arch impressions using the latest hardware and software in different age groups with partially edentulous and fully dentate mandibular jaws.

The null hypotheses investigated were as follows: there are no significant differences between young and elderly subjects in different clinical situations (I), and there are no significant differences among the six impression techniques investigated (II).

## 2. Materials and Methods

Forty-five participants were included in this clinical study and classified into three groups with different clinical situations as follows:

– Group A: Age 27.3 ± 2.7 years with fully dentate mandibular jaw (n = 15)
– Group B: Age 60.6 ± 8.1 years with fully dentate mandibular jaw (n = 15)
– Group C: Age 65.7 ± 6.2 years with partially edentulous mandibular jaw with unilateral edentulous space and adjacent natural teeth (Kennedy Class III, n = 15).

**Figure 1:**
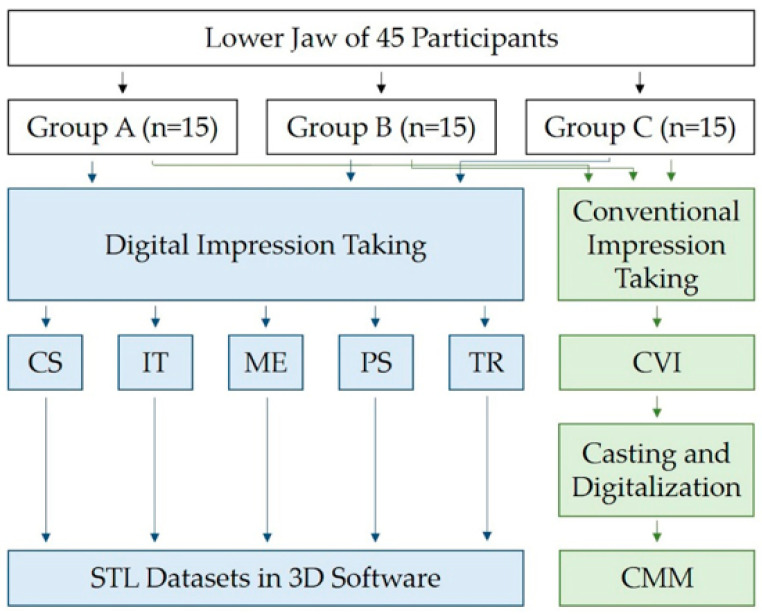
Flow scheme of the clinical trial (CS = CS 3800, IT = iTero Element 5D, ME = Medit i700, PS = Primescan, TR = Trios 4, CVI = conventional impression, STL = standard tessellation language, CMM = coordinate measuring machine).

Good oral hygiene and stable positioning of the reference aid on the occlusal surfaces of the mandibular jaw were defined as further inclusion criteria. Participants with severe systemic disease, epilepsy, or allergies to the materials used were excluded. Furthermore, patients with attachments on tooth surface (e.g., orthodontic appliances) were not included. For a better overview, Figure 1 displays a flow scheme of the clinical trial.

To ensure comparable testing conditions, all experiments were performed by a single operator (J.M.S.) trained on conventional impression taking and all IOSs used in this study.

The investigations were conducted at the Department of Prosthodontics of the Justus Liebig University (JLU) Giessen, Germany, in full compliance with ethical principles, including the Declaration of Helsinki of the World Medical Association. The clinical study was approved by the local ethics committee of the JLU (Ref. no. 163/15) and recorded in the German Clinical Trial Register (DRKS00027135).

**Figure 2:**
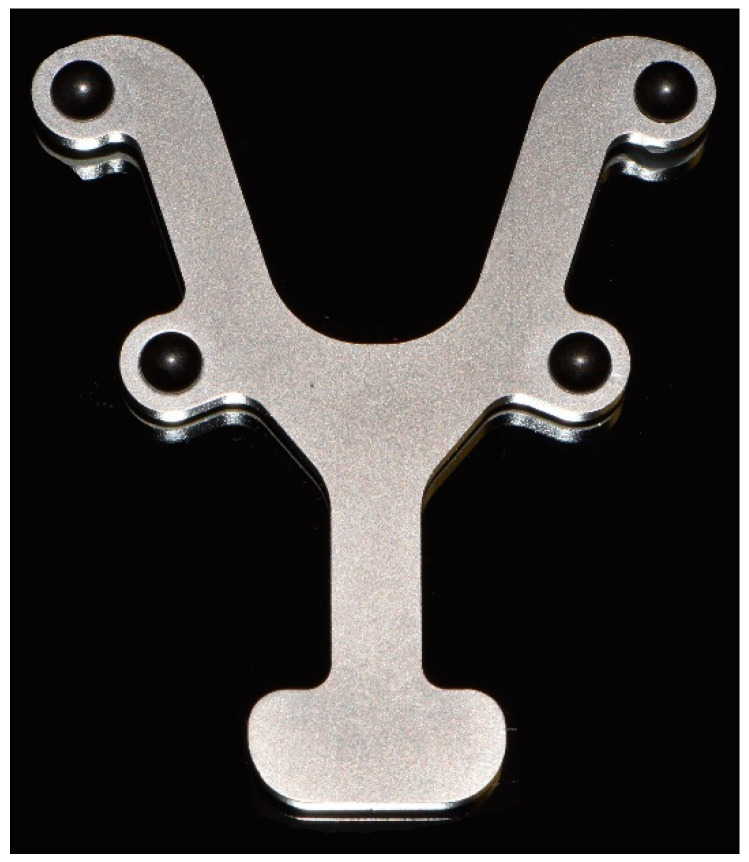
Metal reference guide with the four steel spheres inserted.

According to an established reference method previously described in the literature, four steel spheres (1.3505 100Cr6 DIN5401; TIS GmbH, Gauting, Germany; diameter, 5 mm; roundness, 5000 ± 5.63 μm [22]) were reversibly bonded to the mandibular teeth with a flowable composite (Grandio Flow, Voco, Cuxhaven, Germany) [16,20]. A metal reference guide (Bretthauer GmbH, Dillenburg, Germany; Figure 2) was used to position the spheres presenting a reproducible placement with a precision of <10 μm [23]. When the reference plate was removed, the spheres remained in a defined position, allowing subsequent comparison to the original position in the reference plate.

Before taking digital impressions with the IOS, calibration of the scanner tip with the respective calibration device was applied [24]. The established scan strategy—starting on the occlusal surface, followed by the oral surfaces, and finishing on the buccal surfaces—as recommended by manufacturers was performed [3,16,21]. The IOS used with the corresponding software versions are listed in Table 1.

Cheek retractors (Optragate, Ivoclar Vivadent, Schaan, Lichtenstein) and dry tips (Microbrush International, Grafton, WI, USA) were placed intraorally to control the soft tissue and saliva. Furthermore, uniform light conditions were applied during digital impression taking [25]. For each subject, one scan was performed. Scan data were exported as standard tessellation language (STL) datasets. After completing the digital impressions, the cheek retractor and dry tips were removed, and a CVI was obtained using medium-weight polyether impression material (Impregum Penta Soft Quick, batch number 4811262, 3M Espe, Minneapolis, MN, USA) and a standard metal tray (Ehricke stainless steel, Orbis Dental, Münster, Germany). Before casting with type IV dental stone (Fujirock EP, batch number 1810031, GC Corporation, Tokyo, Japan), the CVI was stored for at least 2 h to ensure elastic recovery.

**Figure 3:**
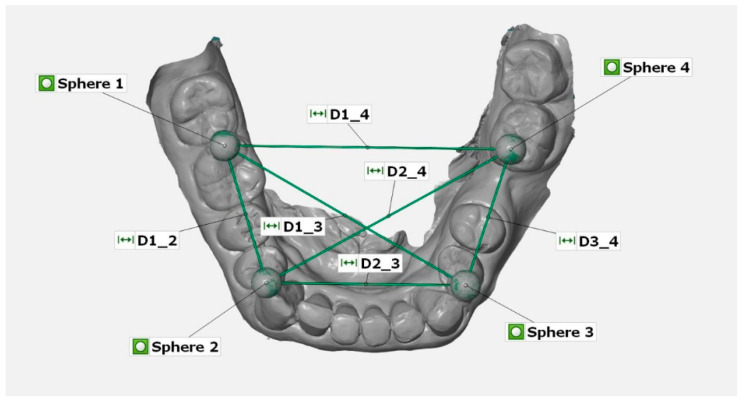
Example of the measurement of linear distances (D1_2, D1_3, D1_4, D2_3, D2_4, D3_4) between the centers of the four spheres 1–4 (top view of STL dataset in GOM software).

Plaster casts were stored under laboratory conditions (temperature 23 ± 1 °C; humidity 50 ± 10%) for at least 5 days before measurement. To measure the reference and plaster models, a co-ordinate measuring machine (CMM, Thome Präzision GmbH, Messel, Germany) with the corresponding software (X4 V10 GA ×64, Metrologic Group, Meylan, France) was used. For the reference dataset the spheres were inserted into the reference aid, measured 10 times with the CMM, and the mean value for each sphere position was calculated. The resulting digital reference model was saved in IGES (Initial Graphics Exchange Specification) format. Subsequently, plaster models of CVIs were also measured with the CMM and saved as digital datasets. The STL datasets of the digital impressions were imported into a three-dimensional analysis software (GOM Inspect 2019, v2.0.1, gom, Braunschweig, Germany). Then, the linear distances between the centers of the spheres were determined (Figure 3).

To measure the deviations between the reference dataset and the models, the reference dataset of the reference aid was imported and saved as computer-aided design data in the analysis program. The scans were imported as an STL dataset and saved as the actual data. Then, fitting elements (Gauss best fit, 3 sigma) were used to construct the sphere elements on the scanned spheres. Subsequently, deviations between the measured distances of the intraoral scans and the reference guide were calculated.

Statistical analysis was performed using the SPSS software (version 28, IBM, Armonk, NY, USA). For trueness [6], the data were transformed using a square root transformation. A three-factor analysis of variance (ANOVA) was performed with the factors’ impression, distance, and dentition. Because impression and distance are repeated factors, dependencies arose, which were considered by a variance component model (procedure MIXED). Distance and impression were modeled as repeated-measures factors; therefore, variance heterogeneity resulting from these factors was also considered. To account for this variance heterogeneity, the three factors were modeled as repeated measurements. The decision criterion was the *p*-value of the interaction, followed by that of the model comparison using -2LL-chi-squared tests. Pairwise comparisons of the hypotheses were requested via the estimated marginal means (margins) and corrected with the Bonferroni correction for multiple pairwise tests. For a better overview, the data are presented in boxplots. For precision, the scatter of different factor levels was tested for homogeneity. Pairwise Levene tests were used to compare impressions within and between groups with respect to distance. To account for the dependencies in the data due to multiple measurements, tests were performed using model residuals. The tests were performed on model residuals from the mixed linear models. The robust Levene tests were based on the medians (Brown–Forsythe test). Differences with *p* < 0.05 were considered statistically significant.

## 3. Results

**Figure 4:**
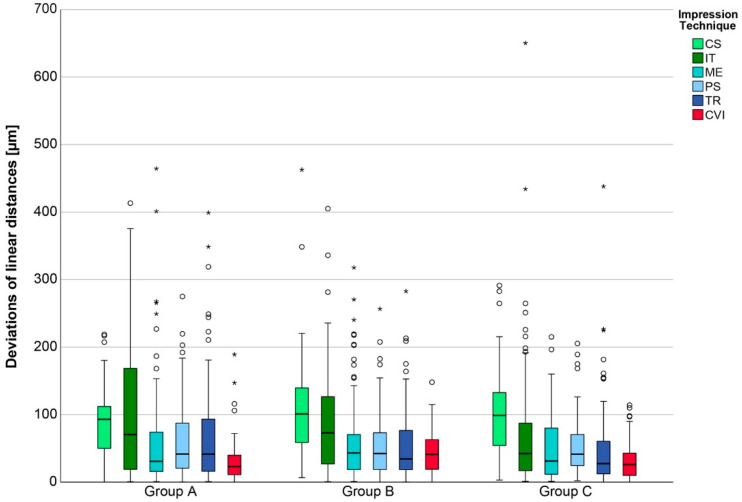
Boxplot diagram of pooled data of the deviations of linear distances for the six impression techniques (CS = CS 3800, IT = iTero Element 5D, ME = Medit i700, PS = Primescan, TR = Trios 4, CVI = conventional impression) classified to group A, B, and C; outliners (O), extreme values (*).

The overall results with pooled data of linear distances for the six impression techniques classified into three groups A, B, and C, are displayed in Figure 4.

Regarding participants’ age, no significant differences between the three groups were observed for IOS in terms of trueness. These findings were confirmed with respect to precision, except for Trios 4, with significant differences between groups A/B and B/C. In contrast to the IOS, the CVI showed significant differences between groups A/B and B/C for trueness and between groups B/C for precision. Table 2 reports the pairwise comparisons for different groups and impression techniques.

Concerning the impression technique, no significant difference was observed between the IOSs ME, PS, and TR compared to the CVI in the overall view. However, the CVI still showed the lowest deviation, especially with respect to long-span distances. The two IOSs, CS and IT, exhibited the highest deviations.

**Figure 5:**
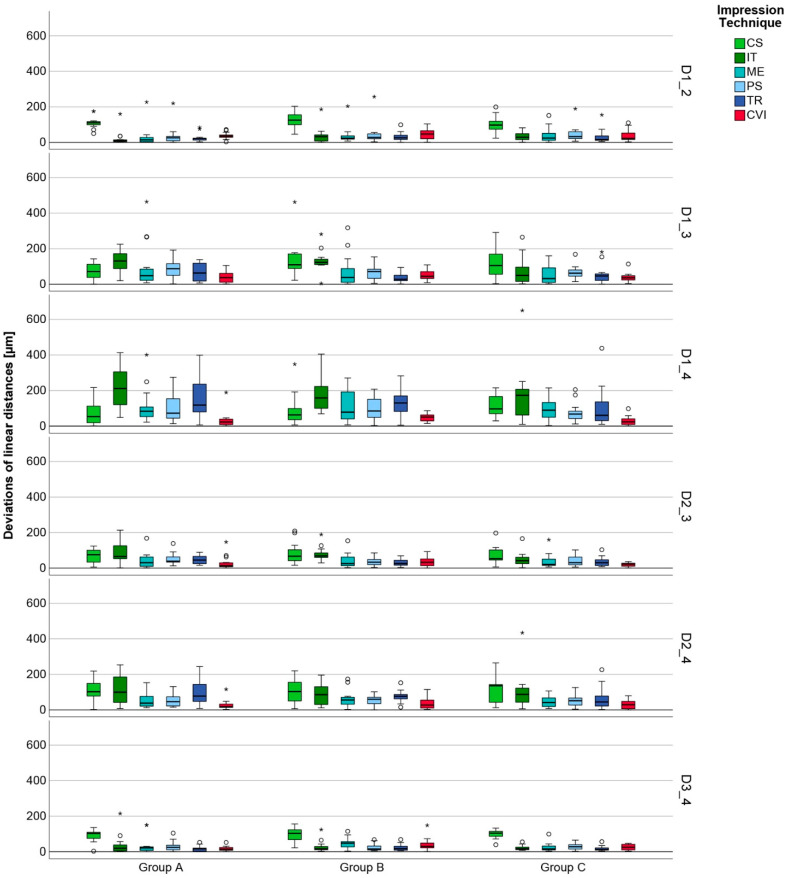
Boxplot diagram of the deviations of the linear distances (D1_2, D1_3, D1_4, D2_3, D2_4, D3_4) in group A, B, and C for the six techniques (CS = CS 3800, IT = iTero Element 5D, ME = Medit i700, PS = Primescan, TR = Trios 4, CVI = conventional impression); outliners (o), extreme values (*).

However, the highest linear deviations were still observed for long-span distances across all the IOSs. Even though the overall results did not show any significant difference in terms of trueness and only a few regarding precision, the detailed analysis of the linear distances exhibited isolated significant differences for accuracy in groups A, B, and C (Figure 5).

Regarding group A, isolated significant differences were observed between the different IOSs for all distances for trueness. In contrast, only considerably fewer significant deviations occurred with precision.

In group B, isolated significant differences between the individual IOSs with respect to distances in terms of trueness were observed as well. In terms of precision, less significant differences were observed between the individual IOSs.

Isolated significant differences between the individual IOSs with respect to distances in terms of trueness were noted in group C. In terms of precision, less significant differences were observed between the individual IOSs. The detailed values are presented in the Appendix A (Table A1, Table A2 and Table A3).

Partly significant differences with respect to young and elderly subjects, clinical situations, and different impression techniques were noted; hence, both null hypotheses were rejected.

## 4. Discussion

In previous studies, numerous influencing factors have been identified with regard to digital impression taking [26]. Thus, the most recent software versions of the respective IOS were used [27,28,29]. Furthermore, all IOSs were calibrated before each impression was taken according to the manufacturer’s instructions to avoid possible deviations [24]. In addition, measurements were conducted with a reference structure [16,20,23] that allows one to determine trueness and precision [15,19]. This allowed the measurement of the individual linear distances and their possible distance deviations across the entire jaw.

As different scanning paths can lead to different results, the scanning path recommended by the manufacturers was used [30,31]. To avoid the influence of different examiners, all impressions were obtained by a trained operator [32]. Due to the methodology used of the reference plate, only impressions of the mandibular jaw were investigated, and this may be regarded as a limitation.

Previous studies addressing the accuracy have typically examined eugnathia dentitions [15,16,20]. However, the dental status and mucosal situation changes with age. Particularly, the mucosal situation in older patients is different from that in young patients. This is directly related to the increase in undercuts and root surfaces being exposed with advancing age on the remaining teeth in the oral cavity [7,8]. To date, only one clinical study has investigated impressions of periodontal compromised dentitions [3]. The increase in the number of undercuts on natural teeth is particularly important for both conventional and digital impressions. While conventional impressions allow the impression material to flow into the undercuts, which typically tear off during removal, high tear strength is often relied upon when selecting the material [33]. However, digital impressions seem to show a clear advantage over conventional impressions, and undercuts also present a particular challenge for the acquisition of a digital impression through the IOS. Because IOSs can only record data in the scanning field, the scanner‘s handpiece must be rotated into the undercuts to detect them as well [3,34]. For this reason, aged dentitions, especially ones with undercuts, make it challenging for the practitioner and the impression method used to obtain high accuracies with regard to the transfer of the intraoral to the model situation.

This is also aggravated by matching and stitching errors predominantly occurring in digital impression taking when long distances and edentulous areas are recorded. Therefore, it was anticipated that dentitions with gaps show higher inaccuracies in contrast to fully dentate jaws because the respective teeth, which typically serve as references, are missing. The comparison of the results of the present study to data in the literature was difficult, since, to our knowledge, only one in vitro study by Kontis et al. [21] has been conducted to date regarding the accuracy of the IOS with missing teeth and a reference structure. It should be noted, however, that owing to the different design of the study (bar versus spheres), only the intermolar distance of the present study could be used for direct comparison.

Regarding the deviations of the individual scanners in the respective groups, significant differences were only found for Trios 4 with regard to precision and CVI with regard to both trueness and precision. The precision of Trios 4 was the lowest in group A. However, compared to Kontis et al. [21], lower deviations were obtained in the present study with Primescan. This might be attributed to the different evaluation and reference methods used. The high inaccuracies of gap situations described by Kontis et al. are supposed to be related to wider gaps, which foster matching or stitching errors [17,19,20,23,35].

However, the results of CVI in the present study could be compared with those in previous studies with the same methodology [16,20]. The results of Trios 4 and Primescan are comparable to a previous study as well [16].

Keul and Güth have used an older version of the iTero IOS [15]. The slightly better results shown by Keul and Güth are supposed to be related on the references bar, which allows a better overlay of the individual datasets. Additionally, in contrast to the investigation by Keul and Güth [15], this study used mandibular jaws. In contrast to the upper jaws and even an in vitro experiment, greater deviations due to the saliva, reflections, and movements of the subjects were expected in the mandibular jaw. Nevertheless, this type of study reflects daily practice since the clinical framework conditions pose challenges to every practitioner.

Unfortunately, currently, no comparable data for the current IOS CS 3800, iTero Element 5D, and Medit i700 exist, which makes it difficult to compare the available results. What was striking in the comparison, however, was that the CS 3800, in contrast to all other IOSs, displayed comparably higher inaccuracies, especially for short distances, regardless of the group.

For CVI, groups A and C did not differ significantly in terms of trueness. In contrast, group B showed greater deviations. However, these were still the smallest compared to the digital impressions. In terms of precision, this was only the case between groups B and C. In principle, the results of the conventional impression could be directly compared to the results of the previous study with regard to group A [16]. A lower trueness in group B was noticeable. This group of subjects with older dentition situations showed the undercuts exactly where possible tear-out distortions could lead to higher deviations. This would also explain the higher trueness in group C, as lower removal forces were necessary when removing the impressions with lower residual tooth stock, which could correlate to lower stresses within the material in connection with the lower necessity of the restoring forces [36].

In summary, only one IOS showed a difference among the different age groups in terms of accuracy. Significant differences were observed only in the CVI. Follow-up studies with participants of an increasingly older population and not limited to young individuals are necessary.

## 5. Conclusions

Within the limitations of this study, we concluded that the latest IOSs still showed limitations in the accuracy of full-arch impressions, even though they all revealed a mean of less than 100 µm deviations on overall view. Furthermore, it has to be noticed that there are still significant differences between the various IOSs. However, they seemed to be unaffected by age and fully dentate or partially edentulous dentitions with small gaps.

## Figures and Tables

**Table 1:** Intraoral scanners used in this study.

Product Name	Manufacturer	Software Version	Abbreviation
CS 3800	Carestream Dental (Atlanta, GA, USA)	1.0.4	CS
iTero Element 5D	Align Technology (San José, CA, USA)	2.7.0.990	IT
Medit i700	Medit (Seoul, South Korea)	1.7.4	ME
Primescan	Dentsply Sirona (Bensheim, Germany)	5.1.3	PS
Trios 4 wireless POD	3Shape (Copenhagen, Denmark)	21.2.0	TR

**Table 2:** Deviations (mean ± standard deviation (SD) [µm]) of the pooled data of linear distances (D1_2, D1_3, D1_4, D2_3, D2_4, D3_4) of six impression techniques (CS = CS 3800, IT = iTero Element 5D, ME = Medit i700, PS = Primescan, TR = Trios 4, CVI = conventional impression) for all groups and statistical analysis for trueness (upper right part) and precision (lower left part, presented in bold type) according to ISO 5725 [6].

Impression Technique	Group	Mean (Trueness) ± SD (Precision) [µm]	Group A	Group B	Group C
CS	A	87.1 ± 51.6	-	0.237	0.533
	B	107.3 ± 71.1	**0.374**	-	>0.999
	C	102.2 ± 60.8	**0.108**	**0.639**	-
IT	A	101.5 ± 97.1	-	>0.999	0.784
	B	90.5 ± 78.3	**0.118**	-	0.502
	C	75.0 ± 96.4	**0.986**	**0.163**	-
ME	A	61.5 ± 81.7	-	0.881	>0.999
	B	62.2 ± 66.0	**0.695**	-	0.314
	C	49.9 ± 48.4	**0.187**	**0.295**	-
PS	A	60.7 ± 55.1	-	0.649	>0.999
	B	53.6 ± 49.6	**0.302**	-	>0.999
	C	52.2 ± 40.4	**0.475**	**0.723**	-
TR	A	69.4 ± 79.3	-	>0.999	0.814
	B	55.0 ± 53.4	**0.013**	-	>0.999
	C	50.6 ± 64.3	**0.746**	**0.041**	-
CVI	A	30.5 ± 31.2	-	0.012	>0.999
	B	43.5 ± 30.4	**0.179**	-	0.020
	C	30.3 ± 25.3	**0.397**	**0.009**	-

## Data Availability

The datasets in this article are available from the corresponding author upon a reasonable request.

## Appendix A

**Table A1:** Deviations (mean ± standard deviation (SD) [µm]) of the linear distances (D1_2, D1_3, D1_4, D2_3, D2_4, D3_4) of six impression techniques (CS = CS 3800, IT = iTero Element 5D, ME = Medit i700, PS = Primescan, TR = Trios 4, CVI = conventional impression) for Group A (young fully dentate) and statistical analysis for trueness (upper right part) and precision (lower left part, presented in bold type) according to ISO 5725 [6].

Linear Distances	Impression Technique		*p*-Value					
		Mean (Trueness) ± SD (Precision) [µm]	CS	IT	ME	PS	TR	CVI
D1_2	CS	110.9 ± 32.2	-	<0.001	<0.001	<0.001	<0.001	<0.001
	IT	21.2 ± 39.8	**0.682**	-	>0.999	>0.999	>0.999	>0.999
	ME	30.3 ± 56.2	**0.583**	**0.797**	-	>0.999	>0.999	>0.999
	PS	35.6 ± 53.5	**0.199**	**0.330**	**0.528**	-	>0.999	>0.999
	TR	25.0 ± 23.5	**0.399**	**0.321**	**0.353**	**0.108**	-	>0.999
	CVI	37.0 ± 18.1	**0.639**	**0.462**	**0.452**	**0.142**	**0.628**	-
D1_3	CS	73.7 ± 47.6	-	>0.999	0.186	>0.999	0.017	<0.001
	IT	125.9 ± 62.8	**0.393**	-	0.002	0.067	<0.001	<0.001
	ME	100.5 ± 130.2	**0.080**	**0.142**	-	>0.999	>0.999	>0.999
	PS	87.4 ± 50.2	**0.214**	**0.470**	**0.329**	-	0.221	<0.001
	TR	67.4 ± 49.7	**0.455**	**0.842**	**0.121**	**0.386**	-	>0.999
	CVI	37.9 ± 31.4	**0.626**	**0.225**	**0.061**	**0.144**	**0.247**	-
D1_4	CS	75.3 ± 69.6	-	<0.001	>0.999	>0.999	0.300	<0.001
	IT	219.9 ± 116.9	**0.098**	-	<0.001	<0.001	0.242	<0.001
	ME	110.5 ± 100.4	**0.343**	**0.518**	-	>0.999	>0.999	<0.001
	PS	104.1 ± 76.4	**0.323**	**0.021**	**0.102**	-	0.823	<0.001
	TR	163.9 ± 122.9	**0.367**	**0.306**	**0.781**	**0.054**	-	<0.001
	CVI	33.2 ± 46.0	**0.010**	**0.002**	**0.009**	**0.031**	**<0.001**	-
D2_3	CS	64.7 ± 40.5	-	>0.999	0.095	>0.999	0.227	<0.001
	IT	88.8 ± 56.7	**0.331**	-	0.011	0.176	0.022	<0.001
	ME	42.1 ± 42.8	**0.554**	**0.539**	-	>0.999	>0.999	0.379
	PS	51.4 ± 33.4	**0.274**	**0.123**	**0.108**	-	>0.999	0.001
	TR	47.5 ± 24.6	**0.117**	**0.882**	**0.291**	**0.018**	-	0.006
	CVI	28.9 ± 38.8	**0.154**	**0.090**	**0.054**	**0.773**	**0.009**	-
D2_4	CS	108.6 ± 64.3	-	>0.999	0.009	0.044	>0.999	<0.001
	IT	116.9 ± 83.3	**0.058**	-	0.022	0.084	>0.999	<0.001
	ME	51.9 ± 39.0	**0.810**	**0.046**	-	>0.999	0.098	0.023
	PS	55.8 ± 38.9	**0.167**	**0.005**	**0.316**	-	0.402	0.002
	TR	95.8 ± 71.9	**0.469**	**0.195**	**0.368**	**0.048**	-	<0.001
	CVI	29.1 ± 27.4	**0.022**	**<0.001**	**0.063**	**0.224**	**0.006**	-
D3_4	CS	89.5 ± 32.1	-	<0.001	<0.001	<0.001	<0.001	<0.001
	IT	36.3 ± 55.1	**0.028**	-	>0.999	>0.999	>0.999	>0.999
	ME	33.6 ± 48.3	**0.015**	**0.918**	-	>0.999	>0.999	>0.999
	PS	30.1 ± 29.2	**0.006**	**0.313**	**0.236**	-	>0.999	>0.999
	TR	16.5 ± 16.1	**0.041**	**0.913**	**0.829**	**0.389**	-	>0.999
	CVI	16.9 ± 13.6	**<0.001**	**0.823**	**0.913**	**0.093**	**0.727**	-

**Table A2:** Deviations (mean ± standard deviation (SD) [µm]) of the linear distances (D1_2, D1_3, D1_4, D2_3, D2_4, D3_4) of six impression techniques (CS = CS 3800, IT = iTero Element 5D, ME = Medit i700, PS = Primescan, TR = Trios 4, CVI = conventional impression) for Group B (young fully dentate) and statistical analysis for trueness (upper right part) and precision (lower left part, presented in bold type) according to ISO 5725 [6].

Linear Distances	Impression Technique		*p*-Value					
		Mean (Trueness) ± SD (Precision) [µm]	CS	IT	ME	PS	TR	CVI
D1_2	CS	127.8 ± 46.4	-	<0.001	<0.001	<0.001	<0.001	<0.001
	IT	36.3 ± 45.7	**0.169**	-	>0.999	<0.001	>0.999	0.164
	ME	37.7 ± 48.3	**0.151**	**0.898**	-	>0.999	>0.999	0.700
	PS	44.4 ± 61.2	**0.432**	**0.898**	**0.833**	-	>0.999	0.461
	TR	31.7 ± 24.8	**<0.001**	**0.003**	**0.010**	**0.083**	-	0.004
	CVI	47.3 ± 33.5	**0.059**	**0.637**	**0.756**	**0.672**	**0.005**	-
D1_3	CS	137.2 ± 100.7	-	>0.999	0.079	0.038	<0.001	<0.001
	IT	132.1 ± 57.9	**0.129**	-	0.011	0.003	<0.001	<0.001
	ME	72.2 ± 91.4	**0.897**	**0.124**	-	>0.999	>0.999	>0.999
	PS	66.3 ± 45.5	**0.263**	**0.511**	**0.277**	-	0.508	>0.999
	TR	36.6 ± 29.2	**0.041**	**0.416**	**0.030**	**0.099**	-	>0.999
	CVI	51.0 ± 28.2	**0.036**	**0.354**	**0.026**	**0.082**	**0.775**	-
D1_4	CS	89.1 ± 88.0	-	<0.001	>0.999	>0.999	>0.999	<0.001
	IT	180.3 ± 95.5	**0.621**	-	0.004	<0.001	0.059	<0.001
	ME	114.2 ± 87.4	**0.565**	**0.904**	-	>0.999	>0.999	<0.001
	PS	97.5 ± 63.7	**0.107**	**0.012**	**0.018**	-	0.362	0.018
	TR	131.6 ± 72.9	**0.099**	**0.010**	**0.016**	**0.932**	-	<0.001
	CVI	47.7 ± 22.5	**0.017**	**<0.001**	**0.002**	**0.026**	**0.028**	-
D2_3	CS	85.3 ± 57.8	-	>0.999	0.028	0.001	<0.001	<0.001
	IT	79.1 ± 39.5	**0.260**	-	0.041	0.002	<0.001	>0.999
	ME	40.4 ± 41.4	**0.511**	**0.617**	-	>0.999	>0.999	<0.001
	PS	37.4 ± 26.0	**0.012**	**0.244**	**0.067**	-	>0.999	>0.999
	TR	30.5 ± 20.5	**0.025**	**0.383**	**0.125**	**0.468**	-	>0.999
	CVI	36.7 ± 28.5	**0.085**	**0.737**	**0.326**	**0.104**	**0.312**	-
D2_4	CS	107.3 ± 68.6	-	>0.999	0.003	<0.001	0.201	<0.001
	IT	88.3 ± 61.3	**0.863**	-	0.111	0.005	>0.999	<0.001
	ME	62.2 ± 47.2	**0.984**	**0.876**	-	>0.999	>0.999	0.651
	PS	52.7 ± 32.7	**0.005**	**0.008**	**0.004**	-	0.099	>0.999
	TR	76.6 ± 33.0	**<0.001**	**<0.001**	**<0.001**	**0.085**	-	0.005
	CVI	38.3 ± 33.6	**0.006**	**0.009**	**0.005**	**0.972**	**0.088**	-
D3_4	CS	96.9 ± 40.7	-	<0.001	<0.001	<0.001	<0.001	<0.001
	IT	26.8 ± 31.6	**0.459**	-	>0.999	>0.999	>0.999	>0.999
	ME	46.2 ± 31.2	**0.323**	**0.112**	-	0.860	0.045	>0.999
	PS	23.0 ± 19.9	**0.371**	**0.994**	**0.064**	-	>0.999	>0.999
	TR	22.9 ± 19.3	**0.001**	**0.043**	**<0.001**	**0.003**	-	0.060
	CVI	39.8 ± 36.2	**0.298**	**0.803**	**0.060**	**0.758**	**0.062**	-

**Table A3:** Deviations (mean ± standard deviation (SD) [µm]) of the linear distances (D1_2, D1_3, D1_4, D2_3, D2_4, D3_4) of six impression techniques (CS = CS 3800, IT = iTero Element 5D, ME = Medit i700, PS = Primescan, TR = Trios 4, CVI = conventional impression) for Group C (young fully dentate) and statistical analysis for trueness (upper right part) and precision (lower left part, presented in bold type) according to ISO 5725 [6].

Linear Distances	Impression Technique		*p*-Value					
		Mean (Trueness) ± SD (Precision) [µm]	CS	IT	ME	PS	TR	CVI
D1_2	CS	101.0 ± 47.7	-	<0.001	<0.001	<0.001	<0.001	<0.001
	IT	34.4 ± 23.8	**0.320**	-	>0.999	>0.999	>0.999	0.760
	ME	41.2 ± 43.9	**0.761**	**0.364**	-	>0.999	>0.999	>0.999
	PS	46.2 ± 44.8	**0.722**	**0.369**	**0.946**	-	>0.999	>0.999
	TR	32.5 ± 39.4	**0.020**	**0.248**	**0.097**	**0.118**	-	0.795
	CVI	38.8 ± 34.8	**0.784**	**0.370**	**0.975**	**0.922**	**0.094**	-
D1_3	CS	120.8 ± 88.1	-	>0.999	0.007	0.215	<0.001	<0.001
	IT	73.4 ± 78.5	**0.657**	-	0.007	0.197	<0.001	<0.001
	ME	52.8 ± 51.9	**0.588**	**0.942**	-	>0.999	>0.999	>0.999
	PS	66.1 ± 37.3	**0.004**	**0.024**	**0.018**	-	0.063	0.001
	TR	51.9 ± 51.5	**0.149**	**0.340**	**0.353**	**0.196**	-	>0.999
	CVI	38.1 ± 26.1	**<0.001**	**0.006**	**0.004**	**0.196**	**0.062**	-
D1_4	CS	111.5 ± 60.3	-	0.003	>0.999	>0.999	>0.999	<0.001
	IT	170.7 ± 154.8	**0.181**	-	0.003	<0.001	0.309	<0.001
	ME	91.9 ± 64.8	**0.162**	**0.693**	-	>0.999	>0.999	<0.001
	PS	76.1 ± 53.2	**0.428**	**0.073**	**0.030**	-	>0.999	<0.001
	TR	102.1 ± 112.0	**0.464**	**0.454**	**0.610**	**0.167**	-	<0.001
	CVI	29.0 ± 25.7	**0.011**	**0.010**	**<0.001**	**0.020**	**0.010**	-
D2_3	CS	70.0 ± 48.9	-	>0.999	<0.001	0.020	<0.001	<0.001
	IT	48.3 ± 39.8	**0.925**	-	0.025	0.407	0.019	<0.001
	ME	40.1 ± 40.5	**0.903**	**0.837**	-	>0.999	>0.999	0.795
	PS	41.2 ± 32.0	**0.036**	**0.035**	**0.083**	-	>0.999	0.005
	TR	35.9 ± 26.3	**0.034**	**0.034**	**0.081**	**0.995**	-	0.089
	CVI	19.5 ± 11.2	**0.001**	**0.002**	**0.007**	**0.052**	**0.042**	-
D2_4	CS	110.5 ± 72.0	-	>0.999	<0.001	<0.001	0.279	<0.001
	IT	102.2 ± 101.5	**0.646**	-	0.056	0.184	>0.999	<0.001
	ME	47.7 ± 33.2	**0.591**	**0.381**	-	>0.999	0.215	0.117
	PS	53.4 ± 35.4	**0.011**	**0.026**	**0.006**	-	0.729	0.016
	TR	63.6 ± 61.8	**0.256**	**0.196**	**0.438**	**0.051**	-	<0.001
	CVI	31.5 ± 27.9	**<0.001**	**0.004**	**<0.001**	**0.013**	**<0.001**	-
D3_4	CS	99.2 ± 24.7	-	<0.001	<0.001	<0.001	<0.001	<0.001
	IT	20.9 ± 13.5	**0.430**	-	>0.999	>0.999	>0.999	>0.999
	ME	25.8 ± 24.2	**0.438**	**0.909**	-	>0.999	0.509	>0.999
	PS	30.0 ± 19.4	**0.014**	**0.142**	**0.252**	-	0.304	>0.999
	TR	17.3 ± 14.0	**0.334**	**0.118**	**0.166**	**0.001**	-	>0.999
	CVI	24.9 ± 17.2	**0.010**	**0.008**	**0.026**	**<0.001**	**0.051**	-

## References

[1] Wöstmann B., Rosentritt M., Ilie N., Lohbauer U. (2018). Abformmaterialien. Werkstoffkunde in der Zahnmedizin.

[2] Mangano F., Gandolfi A., Luongo G., Logozzo S. (2017). Intraoral scanners in dentistry: A review of the current literature. BMC Oral Health.

[3] Schlenz M.A., Schubert V., Schmidt A., Wöstmann B., Ruf S., Klaus K. (2020). Digital versus Conventional Impression Taking Focusing on Interdental Areas: A Clinical Trial. Int. J. Environ. Res. Public Health.

[4] Jordan R.A., Bodechtel C., Hertrampf K., Hoffmann T., Kocher T., Nitschke I., Schiffner U., Stark H., Zimmer S., Micheelis W. (2014). The Fifth German Oral Health Study (Funfte Deutsche Mundgesundheitsstudie, DMS V)—Rationale, design, and methods. BMC Oral Health.

[5] Carlsson G.E., Omar R. (2006). Trends in prosthodontics. Med. Princ. Pract..

[6] (1994). Accuracy (Trueness and Precision) of Measurement Methods and Results—Part 1: General Principles and Definitions.

[7] Heasman P.A., Ritchie M., Asuni A., Gavillet E., Simonsen J.L., Nyvad B. (2017). Gingival recession and root caries in the ageing population: A critical evaluation of treatments. J. Clin. Periodontol..

[8] McKenna G., Burke F.M. (2010). Age-related oral changes. Dent. Update.

[9] Demmer R.T., Papapanou P.N. (2010). Epidemiologic patterns of chronic and aggressive periodontitis. Periodontol 2000.

[10] Eke P.I., Wei L., Borgnakke W.S., Thornton-Evans G., Zhang X., Lu H., McGuire L.C., Genco R.J. (2016). Periodontitis prevalence in adults >/= 65 years of age, in the USA. Periodontol 2000.

[11] Martinez-Canut P., Carrasquer A., Magan R., Lorca A. (1997). A study on factors associated with pathologic tooth migration. J. Clin. Periodontol..

[12] Brunsvold M.A. (2005). Pathologic tooth migration. J. Periodontol..

[13] Melsen B. (2012). Adult Orthodontics.

[14] Desoutter A., Yusuf Solieman O., Subsol G., Tassery H., Cuisinier F., Fages M. (2017). Method to evaluate the noise of 3D intra-oral scanner. PLoS ONE.

[15] Keul C., Güth J.F. (2020). Accuracy of full-arch digital impressions: An in vitro and in vivo comparison. Clin. Oral Investig..

[16] Schmidt A., Klussmann L., Wöstmann B., Schlenz M.A. (2020). Accuracy of Digital and Conventional Full-Arch Impressions in Patients: An Update. J. Clin. Med..

[17] Schmidt A., Billig J.W., Schlenz M.A., Wöstmann B. (2021). The Influence of Using Different Types of Scan Bodies on the Transfer Accuracy of Implant Position: An In Vitro Study. Int. J. Prosthodont..

[18] Giachetti L., Sarti C., Cinelli F., Russo D.S. (2020). Accuracy of Digital Impressions in Fixed Prosthodontics: A Systematic Review of Clinical Studies. Int. J. Prosthodont..

[19] Güth J.F., Edelhoff D., Schweiger J., Keul C. (2016). A new method for the evaluation of the accuracy of full-arch digital impressions in vitro. Clin. Oral Investig..

[20] Kuhr F., Schmidt A., Rehmann P., Wöstmann B. (2016). A new method for assessing the accuracy of full arch impressions in patients. J. Dent..

[21] Kontis P., Guth J.F., Keul C. (2022). Accuracy of full-arch digitalization for partially edentulous jaws—A laboratory study on basis of coordinate-based data analysis. Clin. Oral Investig..

[22] (2014). Rolling Bearings—Balls—Part I: Steel Balls.

[23] Schmidt A., Klussmann L., Schlenz M.A., Wostmann B. (2021). Elastic deformation of the mandibular jaw revisited-a clinical comparison between digital and conventional impressions using a reference. Clin. Oral Investig..

[24] Rehmann P., Sichwardt V., Wöstmann B. (2017). Intraoral Scanning Systems: Need for Maintenance. Int. J. Prosthodont..

[25] Arakida T., Kanazawa M., Iwaki M., Suzuki T., Minakuchi S. (2018). Evaluating the influence of ambient light on scanning trueness, precision, and time of intra oral scanner. J. Prosthodont. Res..

[26] Abduo J., Elseyoufi M. (2018). Accuracy of Intraoral Scanners: A Systematic Review of Influencing Factors. Eur. J. Prosthodont. Restor. Dent..

[27] Schmidt A., Schlenz M.A., Liu H., Kämpe H.S., Wöstmann B. (2021). The Influence of Hard- and Software Improvement of Intraoral Scanners on the Implant Transfer Accuracy from 2012 to 2021: An In Vitro Study. Appl. Sci..

[28] Logozzo S., Zanetti E.M., Franceschini G., Kilpelä A., Mäkynen A. (2014). Recent advances in dental optics—Part I: 3D intraoral scanners for restorative dentistry. Opt. Lasers Eng..

[29] Schmidt A., Benedickt C.R., Schlenz M.A., Rehmann P., Wostmann B. (2020). Torsion and linear accuracy in intraoral scans obtained with different scanning principles. J. Prosthodont. Res..

[30] Ender A., Mehl A. (2013). Influence of scanning strategies on the accuracy of digital intraoral scanning systems. Int. J. Comput. Dent..

[31] Müller P., Ender A., Joda T., Katsoulis J. (2016). Impact of digital intraoral scan strategies on the impression accuracy using the TRIOS Pod scanner. Quintessence Int..

[32] Resende C.C.D., Barbosa T.A.Q., Moura G.F., Tavares L.D.N., Rizzante F.A.P., George F.M., Neves F.D.D., Mendonca G. (2021). Influence of operator experience, scanner type, and scan size on 3D scans. J. Prosthet. Dent..

[33] Lawson N.C., Burgess J.O., Litaker M. (2008). Tear strength of five elastomeric impression materials at two setting times and two tearing rates. J. Esthet. Restor. Dent..

[34] Schubert O., Erdelt K.J., Tittenhofer R., Hajto J., Bergmann A., Guth J.F. (2020). Influence of intraoral scanning on the quality of preparations for all-ceramic single crowns. Clin. Oral Investig..

[35] Park S., Kang H.C., Lee J., Shin J., Shin Y.G. (2015). An enhanced method for registration of dental surfaces partially scanned by a 3D dental laser scanning. Comput. Methods Programs Biomed..

[36] Walker M.P., Alderman N., Petrie C.S., Melander J., McGuire J. (2013). Correlation of impression removal force with elastomeric impression material rigidity and hardness. J. Prosthodont..

